# A Laser Reduced Graphene Oxide Grid Electrode for the Voltammetric Determination of Carbaryl

**DOI:** 10.3390/molecules26165050

**Published:** 2021-08-20

**Authors:** Muhammad Saqib, Elena V. Dorozhko, Jiri Barek, Vlastimil Vyskocil, Elena I. Korotkova, Anastasiia V. Shabalina

**Affiliations:** 1 Department of Chemical Engineering, School of Earth Sciences and Engineering, National Research Tomsk Polytechnic University, Lenin Avenue 30, 634050 Tomsk, Russia; ms.engr9@gmail.com (M.S.); elena-dorozhko@yandex.ru (E.V.D.); eikor@tpu.ru (E.I.K.); 2 UNESCO Laboratory of Environmental Electrochemistry, Department of Analytical Chemistry, Faculty of Science, Charles University in Prague, Albertov 6, CZ-12843 Prague 2, Czech Republic; vyskocil@natur.cuni.cz; 3 Laboratory of Physical and Chemical Method of Analysis, National Research Tomsk State University, Lenin Avenue 36, 634050 Tomsk, Russia; shabalinaav@gmail.com

**Keywords:** laser reduced graphene oxide, grid electrode, graphene oxide, linear sweep voltammetry, carbaryl, pesticides

## Abstract

Laser-reduced graphene oxide (LRGO) on a polyethylene terephthalate (PET) substrate was prepared in one step to obtain the LRGO grid electrode for sensitive carbaryl determination. The grid form results in a grid distribution of different electrochemically active zones affecting the electroactive substance diffusion towards the electrode surface and increasing the electrochemical sensitivity for carbaryl determination. Carbaryl is electrochemically irreversibly oxidized at the secondary amine moiety of the molecule with the loss of one proton and one electron in the pH range from 5 to 7 by linear scan voltammetry (LSV) on the LRGO grid electrode with a scan rate of 300 mV/s. Some interference of the juice matrix molecules does not significantly affect the LSV oxidation current of carbaryl on the LRGO grid electrode after adsorptive accumulation without applied potential. The LRGO grid electrode can be used for LSV determination of carbaryl in fruit juices in the concentration range from 0.25 to 128 mg/L with LOD of 0.1 mg/L. The fabrication of the LRGO grid electrode opens up possibilities for further inexpensive monitoring of carbaryl in other fruit juices and fruits

## 1. Introduction

Pesticides have greatly enhanced crop productivity. However, their widespread application has resulted in the undesirable contamination of the environment. In particular, pesticide contamination of water has become a serious environmental problem in the last few decades. Indeed, the long-term impacts of contaminated water on human health as well as other species are of great concern [1].

**Figure 1:**
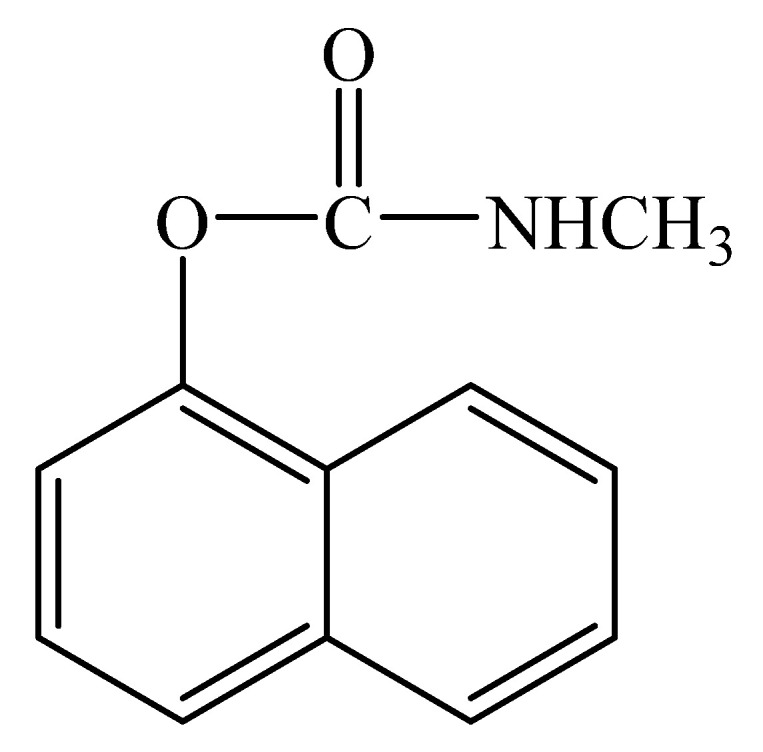
Structural formula of carbaryl.

Carbaryl (Figure 1) is a carbamate insecticide applied to crops which has been classified as a possible carcinogen by the United States Environmental Protection Agency [2]. Carbaryl can harm the human immune system, central nervous system, and endocrine system. According to the United States National Water-Quality Assessment Program, carbaryl is one of the most commonly identified insecticides in water [3] and thus it can negatively affect aquatic organisms as well as humans [2]. In Russia, the carbaryl levels are allowed in the range of 0.002−2.0 mg/kg in milk products, maize, and grains. According to European regulations, the maximum residue level (MRL) for carbaryl is 10 µg/kg for apples, potatoes, and tomatoes, and 50 µg/kg for strawberries [4].

There are several methods for the determination of carbaryl such as differential pulse voltammetry (DPV) [5,6], linear sweep anodic stripping voltammetry [7], colorimetry [8], chronoamperometry [9], fluorimetry [10], high-performance liquid chromatography [11], and gas chromatography [12]. Chromatographic methods are usually time-consuming, expensive, and somewhat complicated. Therefore, electrochemical techniques attract interest because they are simple, cost-effective, and reasonably sensitive and selective [13]. Moreover, their performance can be substantially improved by working electrode modification with various nanoparticles and carbon nanostructures [14] (Table 1).

The key to the successful application of electrochemical methods for pesticide detection is an adequate electrode material with low cost, a broad potential window, high signal-to-noise ratio, and good electrical conductivity. Graphene, a two-dimensional carbon material, has attracted increasing attention during recent years because of its excellent properties [22]. Reduced graphene oxide (rGO) is another promising material for electronic devices and sensors for pesticides detection [23]. Carbon nanomaterials are generally used to improve the working electrodes electroanalytical performance [24]. The large surface area combined with graphene’s excellent electrochemical properties increases the electron transfer rate and detection sensitivity [25].

Unfortunately, in most cases, expensive carbon materials decorated with Co, Ag, and Au nanoparticles produced by laborious methods are used for the electrochemical determination of carbaryl in different objects. The situation can be improved by applying laser-reduced graphene oxide to the PET substrate to obtain an LRGO (laser reduced graphene oxide) grid electrode for inexpensive determination of carbaryl. LRGO on PET substrate is recognized as one of the most versatile electrode materials used in electroanalysis [26]. The grid form results in a grid distribution of different electrochemically active zones, which affects the electroactive substance diffusion front to the electrode surface, thus increasing the electrochemical sensitivity. In this paper, the newly prepared LRGO grid electrode was successfully applied for the determination of carbaryl in fresh juices. The sensitivity of this determination was greatly increased by adsorptive accumulation of carbaryl without applied potential (open circuit potential, OCP) at the LRGO grid electrode.

## 2. Results and Discussion

### 2.1. Characterization of the Laser Reduced Graphene Oxide Electrode

**Figure 2:**
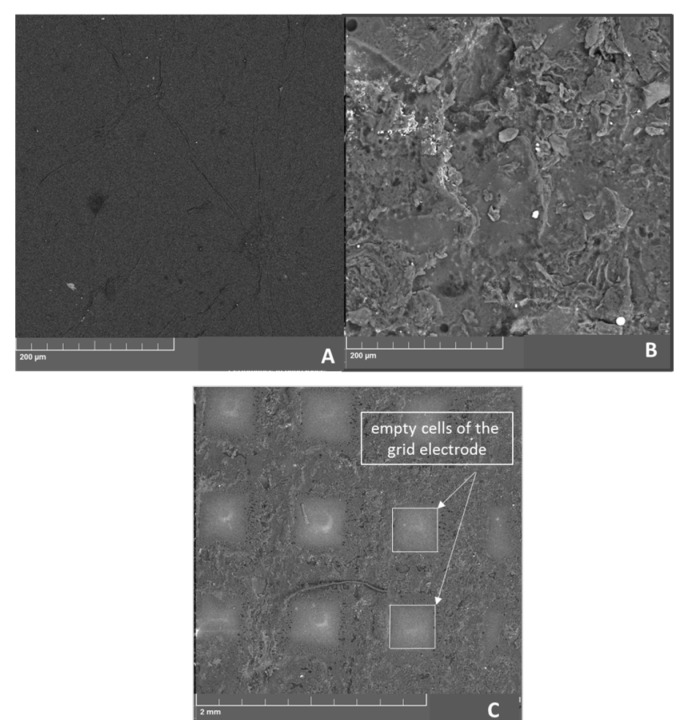
SEM micrographs of (**A**) graphene oxide electrode (**B**) LRGO grid electrode with high resolution (**C**) LRGO gird electrode with low resolution.

The morphological properties of the prepared LRGO electrodes were examined by SEM. Figure 2A shows the SEM images of graphene oxide electrode before laser treatment, which has a smooth surface structure similar to silk. However, reduced graphene oxide electrode prepared by laser treatment shown in Figure 2B displays the expected leaf-like surfaces with wrinkles and folded precincts suitable for the adsorption of carbaryl molecules. Figure 2C shows the LRGO grid electrode on which electroactive micro-sized zones of the LRGO grid and PET zones alternate. These observations can be explained by the presence of sp^3^ carbon atoms and the formation of oxygen-containing functional groups in the basal planes and by various GO structural defects [27]. According to the SEM images in Figure 2, the number of layers reduces and amorphization occurs during graphite oxidation. The degree of aggregation of the LRGO grid electrode is higher than that of the GO electrode, the rGO electrode, and the LRGO planar electrode. The reduction of oxygen-containing functional groups placed in the basal plane of the sp^2^ carbon permits the lamellas of the LRGO sheets to be held together via weak van der Waal forces. Consequently, LRGO sheets are highly aggregated with crumpled features, as shown in their SEM images in Figure 2B [28].

**Figure 3:**
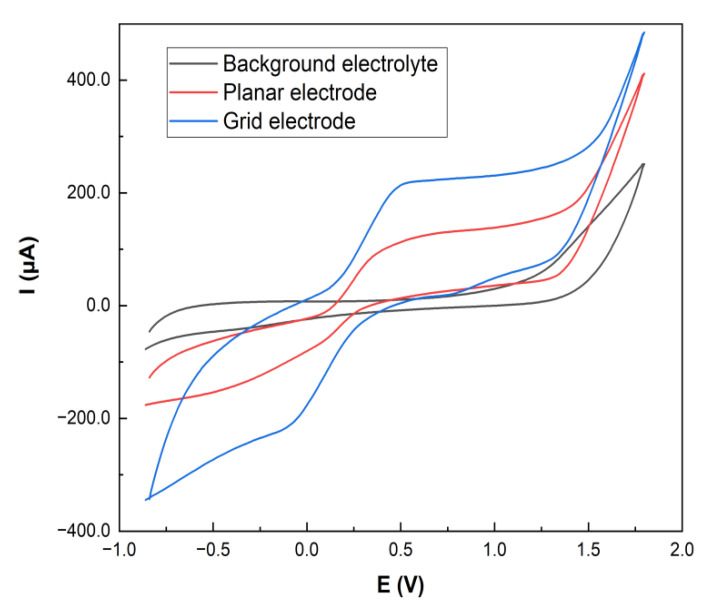
Cyclic voltammograms at 100 mV/s of 0.01 mol/L [Fe(CN)_6_]^3−/4−^ solution in 0.1 mol/L KCl at planar and grid LRGO electrodes.

The electrochemical behavior of the LRGO planar and grid electrodes was investigated by CV using 0.01 mol/L [Fe(CN)_6_]^3−/4−^ in 0.1 M KCl as a probe at 100 mV/s (Figure 3).

**Figure 4:**
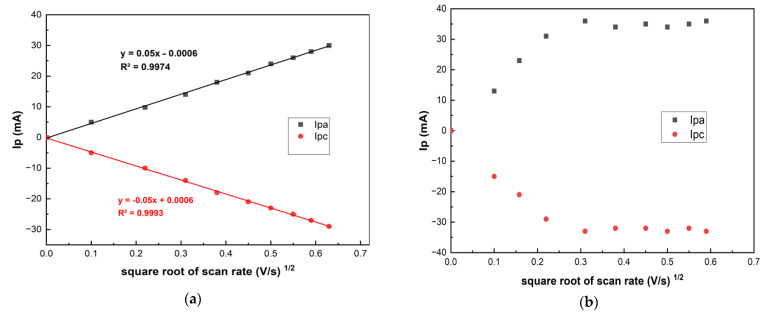
The dependences of the CV redox peak currents of 0.01 mol/L [Fe(CN)_6_]^3−/4−^ in 0.1 mol/L KCI on the square root of scan rate on LRGO planar (**a**) and LRGO grid (**b**) electrodes.

The grid distribution of the zones of different electrochemical activity affects the electroactive substance diffusion front to the electrode surface as well as the electrochemical sensitivity [29]. Figure 3 shows that higher redox currents of [Fe(CN)_6_]^3−/4−^ are observed on the LRGO grid electrode than on the LRGO planar electrode. Figure 4 shows the dependences of the redox currents [Fe(CN)_6_]^3–/4–^ on the square root of scan rate on the LRGO planar (a) and LRGO grid (b) electrodes.

There is a linear dependence of [Fe(CN)_6_]^3−/4−^ redox peak currents on the square root of the scan rate for the LRGO planar electrode, which is typical for diffusion-controlled processes (Figure 4a). For the LRGO grid electrode, the dependence of the redox peak currents on the square root of the scan rate is more complex (Figure 4b) which indicates more complex behavior. This can be explained by the fact that the LRGO planar electrode with planar (linear) diffusion differs from the LRGO grid electrode which is a set of LRGO microelectrodes, each of which enables both radial and linear diffusion. This leads to an increase in redox peak currents of [Fe(CN)_6_]^3−/4−^ at the LRGO grid electrode and the same can be envisaged for other analytes.

### 2.2. Electrochemical Behavior of Carbaryl on the Laser Reduced Graphene Oxide Grid Electrode

**Figure 5:**
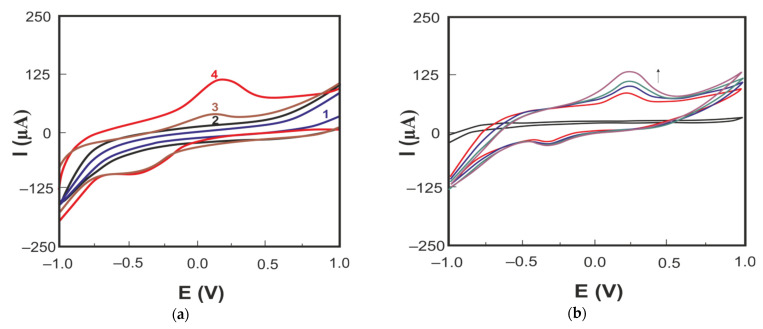
(**a**) Cyclic voltammograms of carbaryl (30 mg/L in 96% ethanol and deionized water (1:1)) on the LRGO plane electrode (curve 3) in the background electrolyte PBS (pH 6.86) (curve 1) and on the LRGO grid electrode (curve 4) in the background electrolyte PBS (pH 6.86) (curve 2); (**b**) cyclic voltammograms of carbaryl (30 mg/L in 96% ethanol and deionized water (1:1)) on the LRGO grid electrode in background electrolyte PBS (pH 6.86) (black curve) at different scan rates (0.2; 0.3; 0.4; 0.5 V/s).

Cyclic voltammograms of carbaryl in PBS (pH 6.86) on LRGO plane (curve 3) and LRGO grid (curve 4) electrode are shown in Figure 5a.

**Figure 6:**
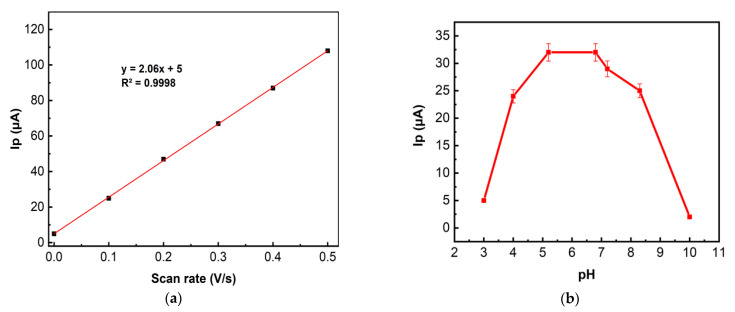
(**a**) The dependence of the carbaryl (15 mg/L in 96% ethanol and deionized water (1:1)) CV anodic peak currents in BR buffer solution (7.00 pH) on the scan rate (0.1 V/s to 0.5 V/s); (**b**) Influence of pH on the LSV anodic peak current of carbaryl (15 mg/L in 96% ethanol and deionized water (1:1)) on the LRGO grid electrode in BR buffers of different pH values; scan rate of 300 mV/s. n = 3

There are well-developed CV oxidation peaks of carbaryl both on the LRGO plane electrode (I_pa_ = 25 μA and E_pa_ = 0.125 V) and on the LRGO grid electrode (I_pa_ = 62 μA, E_pa_ = 0.175 V) (Figure 5a). Lower and more negatively positioned (∆Ep ~ 0.65 V) cathodic peaks nearly independent of the carbaryl concentration confirm irreversible behavior of carbaryl at both electrodes. The anodic CV peak of carbaryl on the LRGO grid electrode is 2.5 times higher than that on the LRGO plane electrode. Therefore, all further studies were carried out using the LRGO grid electrode, since this electrode is a more sensitive sensor for the determination of carbaryl. Figure 5b shows the CV of carbaryl on the LRGO grid electrode at different scan rates. The linear dependence of the anodic peak current on the scan rate (Figure 6a) indicates that the electrode process of carbaryl oxidation is controlled by adsorption. To increase the height of registered carbaryl oxidation signal, the LSV mode was used in further experiments.

The effect of pH on the LSV of carbaryl was evaluated from pH 3.0 to pH 10.0 in BR buffer (see Figure 6b).

Figure 6b shows that the maximum LSV peak current is obtained at pH 5–7. Carbaryl is electrochemically oxidized at the secondary amine moiety of the molecule with the loss of one proton and one electron [15] (see Equation (1))

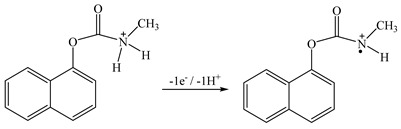
(1)

At low pH, the nitrogen of the carbaryl molecule is protonated, which makes the loss of an electron more difficult, and at a pH above 8 it is hydrolyzed to naphthol analogue, which leads to a sharp decrease in the carbaryl oxidation current [30]. In further studies, PBS (pH 6.86) was used to determine carbaryl.

### 2.3. The Effect of Adsorptive Accumulation Time on the Carbaryl Response on LRGO Grid Electrode

It is known that rGO-based materials are promising for the adsorption of organophosphorus insecticides from aqueous solutions at open circuit potential [31]. This can be the basis of their determination via adsorptive accumulation without applied potential (OCP). This work is the first to report the possibility of using an LRGO grid electrode for LSV of carbaryl in a water-ethanol mixed solution after adsorptive accumulation without applied potential.

**Figure 7:**
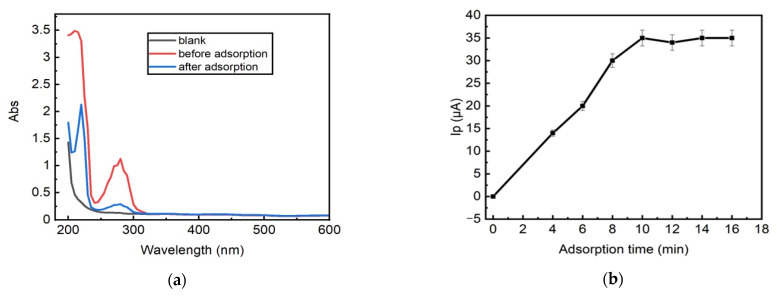
(**a**) UV-vis spectra of carbaryl solution (16 mg/L) in mixture of 96% ethanol and deionized water (1:1) before and after adsorptive accumulation on the LRGO grid electrode. Blank—96% ethanol and deionized water (1:1), measured in 4 μL cuvettes. (**b**) Influence of adsorptive accumulation time on the LSV anodic peak current of carbaryl (16 mg/L) on the LRGO grid electrode.

To prove that the LRGO grid electrode can be used for the adsorptive accumulation of carbaryl, UV–vis spectra of the carbaryl solution before and after adsorption are shown in Figure 7a. The freshly prepared carbaryl (15 mg/L) showed a maximum absorbance at 275 nm, which is the characteristic absorbance peak of carbaryl [32]. After the LRGO grid electrode was immersed into the carbaryl solution for 10 min (maximum adsorption time), the absorbance decreased, indicating the decreased amount of carbaryl in the solution due to adsorption of some carbaryl onto the LRGO grid electrode surface. The measurement of the absorbance of the carbaryl solution before and after adsorptive accumulation on the LRGO grid electrode was carried out by transferring a drop of the solution from the electrode surface to the micro cuvettes (volume of 4 μL) of the spectrophotometer. It should be noted that rGO particles are firmly bounded on LRGO grid electrode and thus do not diffuse into carbaryl solution and do not interfere with carbaryl spectrophotometric monitoring as confirmed by control experiments.

To study the effect of the adsorptive accumulation time without applied potential (OCP) on the LSV oxidation peak height of carbaryl, a 200 μL carbaryl solution (15 mg/L) was dropped onto the LRGO grid electrode surface and kept for various times from 1 to 15 min without applied potential. Before recording the oxidation current by LSV from –0.75 to 1.5 V in PBS (pH 6.86), the electrodes were rinsed with distilled water. The dependence of the peak current of carbaryl oxidation on the time of adsorptive accumulation of carbaryl without applied potential on the LRGO grid electrode is shown in Figure 7b. The optimum time for the adsorptive accumulation of carbaryl on the LRGO grid electrode is 10 min. The pH does not influence the accumulation of carbaryl on the LRGO grid electrode.

The LRGO grid electrode has better detection performance than the planar electrode because of its higher conductivity and higher signal/noise ratio. The alternation of empty cells with protrusions, ribs and vertices of the rGO at the grid electrode after laser treatment allows an increase in the site number with high local surface energy, promoting the adsorption of carbaryl on the grid electrode in comparison with planar electrode.

### 2.4. Analytical Performance

**Figure 8:**
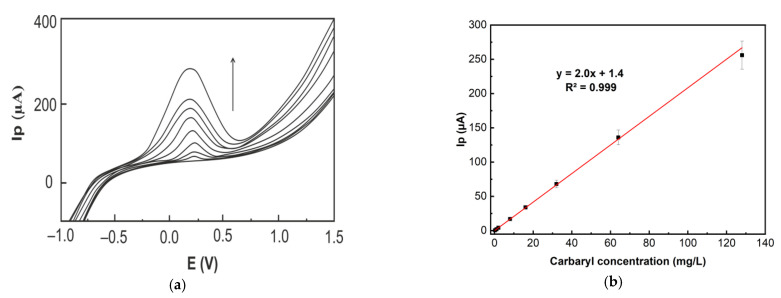
(**a**) Anodic LSVs of carbaryl (increasing concentration 0; 0.25; 1.0; 2.0; 8.0; 16.0; 32.0; 64.0; 128.0 mg/L in 96% ethanol and deionized water (1:1)) at LRGO grid electrode in PBS (pH 6.86) at 300 mV/s. The adsorptive accumulation time of carbaryl from the solution was 10 min. (**b**) Calibration dependence of the LSV anodic peak height on carbaryl concentration.

Figure 8a shows the anodic LSVs of carbaryl after its adsorptive accumulation for 10 min on the surface of the LRGO grid electrode. The LSV peak heights are proportional to the carbaryl concentration in the range from 0.25 to 128.0 mg/L (Figure 8b). A limit of detection (LOD) of 0.1 mg/L was obtained based on 3S/N.

**Figure 9:**
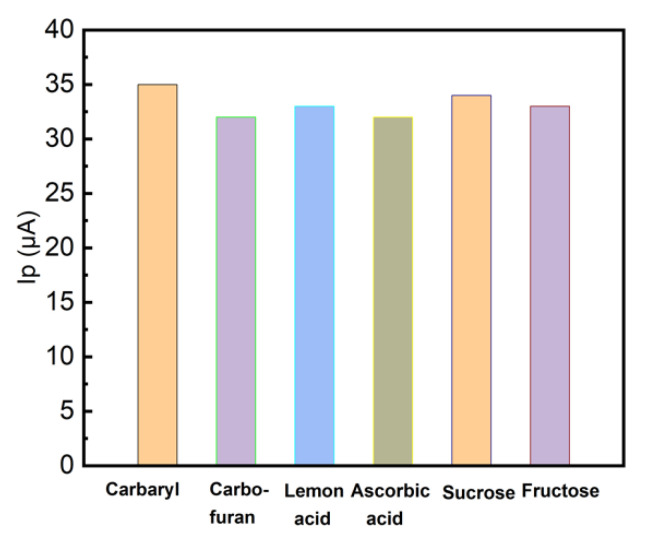
LSV anodic peak height of carbaryl (16 mg/L in 96% ethanol and deionized water (1:1)) at LRGO grid electrode. Adsorptive accumulation for 10 min in PBS (pH 6.86) containing carbaryl in the absence and presence of 10-fold concentration of carbofuran, citric and ascorbic acids, sucrose and fructose, respectively. The scan rate 300 mV/s.

To investigate the LRGO grid electrodes for the determination of carbaryl in fruit juices, we have explored the effects of juice matrix components on the anodic LSV peak current of carbaryl (Figure 9).

The carbaryl concentration was 16 mg/L and carbofuran was chosen as a model interfering component of carbamate nature. When 10-fold amount of carbofuran, citric and ascorbic acid, sucrose and fructose were added, the results were practically unaffected (RSD < 5%), because the direct electrochemical detection of carbaryl utilizes the electrochemical activity of the nitrogen of the naphthyl ester of *N*-methylcarbamic acid. Carbofuran shows its electrochemical activity only after alkaline hydrolysis in the form of carbofuran-phenol [21] and does not significantly affect the determination of carbaryl.

To further demonstrate the practical applicability of the proposed method, a recovery test was performed by adding different amounts of carbaryl (0.25; 31.25; and 125 mg/L) to freshly prepared apple and orange juices, and the results are summarized in Table 2.

The proposed LRGO grid electrodes were successfully applied for the determination of carbaryl in apple and orange juices with favorable recoveries from 92.0% to 107.0%. It is also recommended for the determination of carbaryl in other fruit juices and fruits. The linearity range from 0.25 to 128.0 mg/L for the electrochemical determination of carbaryl in fruits on the LRGO grid electrode is wider, and the limit of detection (LOD) of 0.1 mg/L is comparable with previous studies (Table 1). Moreover, the LRGO grid electrode can be prepared using a simple, convenient, and inexpensive process.

## 3. Materials and Methods

### 3.1. Reagents

A carbaryl standard (99%) and ethanol were purchased from Sigma-Aldrich (Saint Louis, MO, USA). Graphene oxide (4 mg/mL dispersion in water) was purchased from the Graphenea company (www.graphenea.com, accessed on 1 August 2021). The apples and oranges were purchased from the local market in Tomsk, Russia.

### 3.2. Preparation of the Laser Reduced Graphene Oxide Electrode

**Figure 10:**
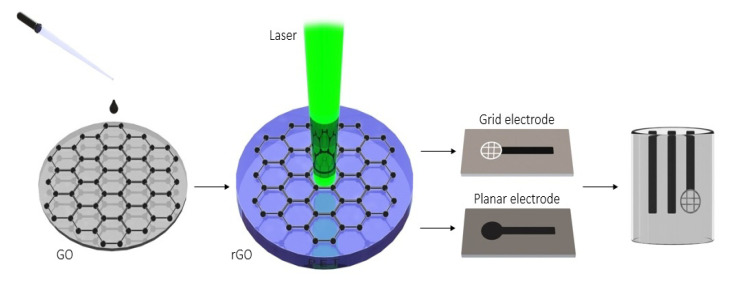
Schematic illustration of the fabrication of laser reduced graphene oxide electrode for the determination of carbaryl.

A polyethylene terephthalate (PET) sheet 5 × 25 mm^2^ in size was used as a substrate for graphene oxide (GO) film deposition by the drop-casting method without further treatment to obtain a homogeneous electrode surface (see Figure 10). The drop casting dispersion volume, concentration and solvent were optimized to obtain the most effective electrodes in terms of sensitivity and reproducibility. Briefly, 100 µL of GO (2 mg/mL) was deposited by drop-casting on the PET surface and dried carefully for 2 h at laboratory temperature (22 ± 2 °C). GO to rGO conversion was performed using a laser engraver with an optimal power (600 mW) at wavelength of 405 nm. There are two types of engraved electrodes: either with planar or with grid geometry. The second one enhances the surface section’s sensitivity to the pesticide, and thus it was used in this paper. The size of the working grid was 12.57 mm^2^. The distance between the electrode and the laser head was 5 mm to prevent burning during the reduction.

### 3.3. Apparatus and Measurements

All electrochemical measurements were conducted on a portable PalmSens 4 Potentiostat analyzer (Palm Instruments BV, Houten, The Netherlands) in a three-electrode arrangement with a LRGO electrode prepared as described above as a working electrode, Ag/AgCl (1 mol/L KCl) as a reference electrode, and a platinum wire as a counter electrode. The structure and morphology of the LRGO electrodes (planar or grid) were investigated by high-resolution scanning electron microscopy (SEM) (Vega 3H, Tescan, Brno, Czech Republic) at Tomsk State University (Tomsk, Russia).

A standard carbaryl solution was prepared by dissolving 0.0040 g in 1 mL of a mixture of 96% ethanol and deionized water (1:1).

Both cyclic voltammetry (CV) and linear scan voltammetry (LSV) were employed for carbaryl detection in the potential range from −1.0 to 1.6 V in 0.1 mol/L phosphate buffer solution (PBS) (pH 6.86). The voltammograms were recorded at a potential scan rate of 300 mV/s unless stated otherwise.

A 0.1 mol/L Britton–Robinson (BR) buffer solution was prepared in the usual way from phosphoric, glacial, acetic, and boric acid and from 1 mol/L NaOH.

Apples and oranges were grated to obtain the juice. Then, 250 µL of apple juice were mixed with the 250 µL of carbaryl standard solution at requested concentrations for further experiments. Orange juice samples were prepared by means of the same procedure. Then, 100 µL of the prepared spiked sample of juice with standard solution of carbaryl (0.25; 31.25; 126.00 mg/L) were deposited on the electrode surface for 10 min using the drop casting method at laboratory conditions.

The electrode was then rinsed three times with PBS, placed into the electrochemical cell containing 8 mL of PBS (pH 6.86) and the carbaryl oxidation signal was recorded by LSV. The limit of detection (LOD) was calculated as 3s/b (where s is the standard deviation of blank and b is the slope of the straight section of the calibration curve) (S/N = 3).

## 4. Conclusions

The laser-reduced graphene oxide on a PET substrate was prepared using a simple, convenient, and inexpensive technique to obtain a laser reduced graphene oxide grid electrode for sensitive carbaryl determination. The thus prepared LRGO grid electrode was used for LSV determination of carbaryl which is electrochemically irreversibly oxidized at the secondary amine moiety of the molecule with the loss of one proton and one electron in the pH range from 5 to 7. Juice matrix components did not significantly affect the LSV oxidation current of carbaryl after its adsorptive accumulation without applied potential for 10 min. The LRGO grid electrode was used for the detection of carbaryl in fruit juices in the concentration range from 0.25 to 128 mg/L with LOD of 0.1 mg/L after its adsorptive accumulation. The anodic LSV peak height of carbaryl on the LRGO grid electrode was proportional to the carbaryl concentration. It was verified that the LRGO grid electrode can be used for direct determination of carbaryl in apple and orange juices as well as other fruit juices and fruits.

## Figures and Tables

**Table 1:** Electrochemical methods for the determination of carbaryl in various matrices.

Method	Electrode	Linear Range (µM)	LOD (µM)	Matrix	Reference
DPV	CoO/rGO/GCE	0.50−200	0.037	Fruits	[5]
SWV	GC/MWCNT/CoPc	0.30−6.61	0.005	River water	[15]
DPV	rGO/Cu/CuO-Ag/GCE	0.05−20.0	0.005	Fruits	[16]
CV	Graphene-modified boron-doped diamond electrode	1−6	0.07	Fruits	[17]
SWV	Graphene oxide-ionic liquid/GCE	0.10–12.0	0.02	Fruits	[14]
AdSV	GCE-CoO/reduced graphene oxide	0.5−200	0.021	Fruits and vegetable	[18]
DPV	GCE-graphene oxide/ionic liquid	0.1−12.0	0.02	Fruits	[19]
CV	GCE modified by MIP decorated by rGO and Au nanoparticles	1−6	0.07	Apple juice	[20]
CV	SPE-carbon black nanoparticle	0.1−100	0.048	Wheat	[21]

DPV: differential pulse voltammetry; SWV: square wave voltammetry; CV: cyclic voltammetry; AdSV: adsorptive stripping voltammetry; rGO: reduced graphene oxide; GCE: glassy carbon electrode; GC/MWCNT/CoPc: multi-walled carbon nanotube/cobalt phthalocyanine modified electrode.

**Table 2:** Recovery study of carbaryl in freshly prepared apple and orange juices (*n* = 3) in PBS (pH 6.86) with adsorptive accumulation time of 10 min and scan rate of 300 mV/s.

Juice Sample	Carbaryl Added (mg/L)	Carbaryl Found (mg/L)	Recovery (%)	RSD (%)
Apple	0.25	0.26	104.0	5.4
	31.25	32.82	105.0	5.0
	126.00	123.50	98.0	4.8
Orange	0.25	0.23	92.0	5.2
	31.25	29.55	94.6	5.0
	126.00	124.00	98.4	4.9

## Data Availability

All relevant data generated during the study are included in this article.

## Sample Availability

The tested samples are available from the authors.
